# Prevalence and Correlates of Dyslipidemia in HIV‐Positive Patients at Selected Public Hospitals in Addis Ababa, Ethiopia, 2025

**DOI:** 10.1155/arat/9582674

**Published:** 2026-08-24

**Authors:** Nebiat Adane, Mekoya Mengistu, Tariku Fekadu, Abraham Tekola Gebremedhn, Mahder Abebe Asres, Nevena Ivanova, Subah Abderehim Yesuf

**Affiliations:** ^1^ Department of Internal Medicine, Yekatit 12 Hospital Medical College, Addis Ababa, Ethiopia; ^2^ Ministry of Health, Armauer Hansen Research Institute, Addis Ababa, Ethiopia, ahri.gov.et; ^3^ Internal Medicine Department, School of Medicine, Addis Ababa University, Addis Ababa, Ethiopia, aau.edu.et; ^4^ Department of Urology and General Medicine, Faculty of Medicine, Medical University of Plovdiv, Plovdiv, Bulgaria, mu-plovdiv.bg; ^5^ St Karidad MHAT, Karidad Medical Health Center, Cardiology, Plovdiv, Bulgaria; ^6^ Department of Internal Diseases, Social Medicine, General Medicine, Physical and Rehabilitation Medicine, and Disaster Medicine, Faculty of Medicine, Burgas State University “Prof. Dr. Asen Zlatarov”, Burgas, Bulgaria; ^7^ Department of Family Medicine, St. Paul’s Hospital Millennium Medical College, Addis Ababa, Ethiopia, sphmmc.edu.et; ^8^ Department of Family Medicine, St. Peter’s Specialized Hospital, Addis Ababa, Ethiopia

**Keywords:** associated factors, dyslipidemia, Ethiopia, HIV/AIDS, prevalence

## Abstract

**Background:**

Dyslipidemia is a common metabolic complication among people living with HIV and a major contributor to cardiovascular morbidity and mortality. However, it remains insufficiently characterized in Ethiopia, particularly in contemporary urban ART settings.

**Methods:**

We conducted a facility‐based cross‐sectional study among 398 patients selected by systematic random sampling from November 1 to December 31, 2025. Data were collected using a structured data collection tool, entered into KoboToolbox, and analyzed in SPSS Version 27. Bivariable and multivariable logistic regression analyses were used to identify factors associated with dyslipidemia. Odds ratios (ORs) with 95% confidence intervals (CIs) were calculated, and *p* < 0.05 was considered statistically significant.

**Results:**

The median age of participants was 50 years (IQR: 42–56), and 66.8% (*n* = 266) were female. The median duration since HIV diagnosis was 19 years (IQR: 15–20). Predominant regimens included TDF+3TC+ DTG (72.8%). The prevalence of dyslipidemia was 65.3% (95% CI: 60.5–70.1), with elevated triglycerides (37.2%), total cholesterol (34.7%), high LDL‐C (34.2%), and low HDL‐C (28.1%). On multivariable analysis, dyslipidemia was significantly associated with history of ART change (AOR 2.62, 95% CI: 1.13–6.04), hypertension (AOR 2.45, 95% CI: 1.28–4.69), overweight (AOR 3.56, 95% CI: 1.44–8.81), obesity (AOR 5.15, 95% CI: 1.16–22.82), and sedentary and moderate physical activity (AOR 9.46, 95% CI: 2.46–36.32 and AOR 3.80, 95% CI: 1.77–8.18, respectively).

**Conclusion:**

Dyslipidemia was prevalent among approximately two in three HIV‐positive patients receiving long‐term ART at two tertiary hospitals in Addis Ababa, Ethiopia. These findings highlight the need for routine lipid monitoring and integrated cardiovascular risk management within HIV care settings in similar urban, tertiary‐care contexts.

## 1. Introduction

Dyslipidemia, characterized by abnormal levels of lipids in the blood—including elevated total cholesterol (TC), low‐density lipoprotein cholesterol (LDL‐C), triglycerides (TG), and/or reduced high‐density lipoprotein cholesterol (HDL‐C)—is a significant risk factor for cardiovascular disease (CVD) globally [1]. It is also a common metabolic complication among people living with HIV (PLHIV) and a key driver of cardiovascular morbidity and mortality in this population [2, 3].

In the context of human immunodeficiency virus (HIV) infection, the prevalence and patterns of dyslipidemia are influenced by both the virus itself and the use of antiretroviral therapy (ART) [2]. HIV induces dyslipidemia via increased hepatic very‐low‐density lipoprotein (VLDL) production, altered cytokine profiles, and impaired lipid clearance. ART also contributes to lipid disturbances, often manifesting as hypertriglyceridemia, reduced HDL‐C, and elevated LDL‐C. Additionally, adipose tissue serves as a viral reservoir for HIV, leading to unfavorable clinical outcomes [3, 4].

The widespread implementation of ART has substantially improved the life expectancy of PLHIV. However, this increased longevity has shifted the disease burden toward noncommunicable conditions, notably CVD [5, 6]. Of particular importance, dyslipidemia is a critical factor in this transition, necessitating a multidisciplinary, proactive management approach as an integral part of comprehensive HIV care to mitigate elevated CVD risk in this population. Effective management strategies include regular lipid monitoring, lifestyle modification, and pharmacological interventions tailored to minimize drug–drug interactions with ART regimens, together with close monitoring for adverse effects [7–10]. However, target lipid control remains suboptimal, reflecting inadequate cardiovascular risk control among PLHIV [11].

The burden of HIV in sub‐Saharan Africa (SSA) remains substantial, with approximately 25.6 million PLHIV in the region as of 2023, representing over two‐thirds of the global total [12]. Previous studies have reported a high prevalence of dyslipidemia among PLHIV in SSA, ranging from approximately 55.2% to over 76.6%, with demographic and clinical factors influencing lipid abnormalities [13].

Several facility‐based studies across regions and recent systematic reviews have examined dyslipidemia among PLHIV in Ethiopia [13, 14]. However, important gaps remain. Most available studies were conducted in single‐center settings or in earlier ART eras, and fewer have assessed dyslipidemia in a contemporary urban ART population with a broader range of clinical and behavioral variables, particularly after the introduction of newer ART regimens such as dolutegravir‐based therapy [13–15] and the broader adoption of the updated 2025 ESC guidance that recommends lower LDL‐C thresholds [16]. Accordingly, comprehensive, updated multicenter studies in Addis Ababa remain limited.

Additionally, standardized protocols for dyslipidemia screening and management in HIV care are not yet uniformly implemented, highlighting the need for evidence‐based guidelines tailored to the Ethiopian context. HIV‐specific lipid targets are absent from national Ethiopian protocols, which largely rely on adapted Western frameworks. Moreover, novel agents remain unavailable in most SSA settings despite their proven efficacy [17]. This multicenter study therefore aims to address those gaps and support integration of cost‐effective dyslipidemia screening and context‐appropriate treatment algorithms into Ethiopian HIV protocols to reduce cardiovascular morbidity.

## 2. Methods and Materials

### 2.1. Study Area and Period

This facility‐based cross‐sectional study was conducted in Addis Ababa, the capital city of Ethiopia, from November 1 to December 31, 2025. Addis Ababa comprises eleven subcities and 117 Woredas and is served by 12 governmental hospitals and approximately 40 private hospitals. The city has a high population density, with an estimated total population of 5,957,000 as of April 2025 [18].

As the largest referral facility in the country, Tikur Anbessa Specialized Hospital (TASH)’s ART clinic manages one of the highest patient volumes, serving over 3200 HIV‐positive adults. Similarly, the corresponding unit of Yekatit 12 Hospital and Medical College (Y12HMC) provides ART care for 3157 individuals aged 18 years or older.

### 2.2. Population

#### 2.2.1. Source Population

The source population comprised HIV‐positive patients receiving care at the ART clinics of the study hospitals—TASH and Y12HMC—in Addis Ababa, Ethiopia.

#### 2.2.2. Study Population

The study population comprised HIV‐positive patients attending the ART clinics at TASH and Y12HMC in Addis Ababa, Ethiopia, during the study period, who were selected using a systematic sampling technique and met the prespecified eligibility criteria.

### 2.3. Eligibility Criteria

#### 2.3.1. Inclusion Criteria

- Adults aged ≥ 18 years with confirmed HIV‐positive status, regardless of lipid‐lowering medication use [19].
- Patients receiving ART for ≥ 6 months [19].
- Availability of recent (≤ 3 months) lipid profile data to reduce temporal bias and ensure the dyslipidemia status is accurately captured at or near the time of study enrollment [19, 20].

#### 2.3.2. Exclusion Criteria

- Pregnancy or lactation (due to physiological lipid changes) [21].

### 2.4. Sample Size Determination

The sample size for this study was determined using the single population proportion formula, *n* = *z* ^2^ *p*(1 − *p*)/*e* ^2^, informed by a prior Central Ethiopian study reporting 55.2% dyslipidemia prevalence among PLHIV [22]. Assumptions included a 95% confidence level (*Z*_*a*/2_ = 1.96), 5% margin of error (*e* = 0.05), and anticipated prevalence (*p* = 0.552), yielding *n* = 380.

This surpassed sample sizes from double population proportion formulas for associated factors, including primary education, body mass index (BMI) > 30 kg/m^2^, and female sex. A 10% nonresponse adjustment to the largest estimate (380) produced the final sample size of 418.

### 2.5. Sampling Procedure

Addis Ababa has 12 public hospitals, from which two (TASH and Y12HMC) were randomly selected via lottery. The ART clinics at these sites followed approximately 3200 (TASH) and 3157 (Y12HMC) adults living with HIV. The sampling frame consisted of all adult HIV patients with clinical appointments during the study period (TASH: 3200; Y12HMC: 3157).

Systematic random sampling was then applied at each site. The sampling interval was *k* = 15 (TASH: 3200 ÷ 210 ≈ 15; Y12HMC: 3157 ÷ 208 ≈ 15). Starting from a randomly selected first patient (via lottery at each clinic register), every 15^th^ consecutive patient was enumerated and included.

### 2.6. Operational Definitions

• **Dyslipidemia**: is defined as the presence of one or more of the following lipid abnormalities, measured from fasting blood samples:
  ⁃ TC ≥ 200 mg/dL [23].
  ⁃ TG ≥ 150 mg/dL [23].
  ⁃ LDL‐C ≥ 115 mg/dL [23].
  ⁃ HDL‐C < 40 mg/dL for men or < 50 mg/dL for women [16, 23].
• **CD4+ T-lymphocyte count**: Number of CD4‐positive T lymphocytes per cubic millimeter of blood (cells/mm^3^), measured by flow cytometry in participating hospital laboratories. Categories (CDC classification):
  ⁃ Advanced immunosuppression (AIDS‐defining): CD4 count < 200 cells/mm^3^
  ⁃ Moderate immunosuppression: CD4 count 200–499 cells/mm^3^
  ⁃ Preserved immune function: CD4 count ≥ 500 cells/mm^3^
• **HIV viral load**: Quantity of HIV‐1 RNA copies per milliliter of plasma (copies/mL), measured by PCR assay in participating hospital laboratories. Dichotomized for viral suppression:
  ⁃ Virologically suppressed (undetectable): viral load < 50 copies/mL
  ⁃ Virologically unsuppressed: viral load ≥ 50 copies/mL
• **ART regimen change**: any switch from one antiretroviral regimen to another, either within‐class or between‐classes, regardless of the underlying reason.
• **BMI**: was calculated as body weight in kilograms divided by height in meters squared (kg/m^2^), BMI was categorized according to the WHO International Classification for adults [24]:
  ⁃ Underweight: BMI < 18.5 kg/m^2^
  ⁃ Normal weight: BMI 18.5–24.9 kg/m^2^
  ⁃ Overweight: BMI 25.0–29.9 kg/m^2^
  ⁃ Obese: BMI ≥ 30.0 kg/m^2^
• **Current smoker**: represents an active smoker or a smoker who quit within the previous 1 year [25].
• **Former smoker**: stands for a smoker who had quit smoking for more than a year before the time of data collection [25].
• **Physical activity**: was assessed using a structured questionnaire adapted from WHO guidelines. Participants were classified into three categories based on weekly activity duration [26]:
  ⁃ Sedentary: < 150 minutes/week of moderate activity (or < 75 minutes/week vigorous)
  ⁃ Moderate: 150–299 minutes/week moderate (or 75–149 minutes/week vigorous)
  ⁃ High: ≥ 300 minutes/week moderate (or ≥ 150 minutes/week vigorous)
• **Occasional alcohol intake**: Consumption of alcohol‐based beverages less than once per week or during specific events such as holidays, ceremonies, or social gatherings.
• **Regular alcohol intake**: Consumption of any type/volume of alcohol‐based beverages (local or commercial) at regular intervals, typically ≥ 1 times per week with consistent frequency.

### 2.7. Data Collection Tools and Techniques

Data were collected using a structured, pretested data collection tool adapted from existing literature [13, 22, 27–31]. The tool was divided into four sections: demographic information, clinical variables, laboratory parameters, and behavioral and lifestyle details. Data were obtained from ART patient registry logbooks and medical records, supplemented by patient interviews when necessary to gather missing behavioral and lifestyle information. Patients without recent lipid profiles were scheduled for fasting lipid panel tests during the study period. Fasting venous blood samples (5 mL) were collected after 8–12 h of overnight fasting. Serum was separated by centrifugation at 3000 rpm for 10 minutes within 2 h of collection. Lipid profiles were measured using automated enzymatic colorimetric methods on automated analyzers at the respective hospital laboratories. All laboratories participated in the Ethiopian External Quality Assurance Scheme (EQAS) for lipid testing, and internal quality control was performed daily.

Two experienced data collectors were recruited and provided with one‐day training on data collection procedures.

### 2.8. Data Processing and Analysis

A comprehensive data quality management strategy was implemented to ensure the reliability and integrity of the study data. Data were coded, entered, and cleaned using KoboToolbox, a digital data collection and form‐management platform for field data entry and quality control. Statistical analyses were performed using IBM SPSS Statistics Version 27. Double data entry by a trained clerk ensured accuracy, followed by cleaning via sorting, frequency distributions, and cross‐tabulations to identify missing values and outliers.

Patient characteristics and dyslipidemia prevalence were summarized descriptively. Continuous variables were expressed as mean ± standard deviation (SD) for normally distributed data (assessed via the Shapiro–Wilk test) or median [interquartile range (IQR)] for non‐normally distributed data. Bivariable screening computed crude odds ratios (ORs) with 95% confidence intervals (CIs), and variables with *p* < 0.25 were selected for multivariable logistic regression. The multivariable model generated adjusted ORs with 95% CIs to identify significant factors associated with dyslipidemia, with *p* < 0.05 considered statistically significant. Collinearity diagnostics were assessed using variance inflation factors (VIFs), with VIF < 10 indicating no substantial multicollinearity. Model goodness‐of‐fit was evaluated using the Hosmer–Lemeshow test (*χ* ^2^ = 9.3; *p* = 0.318).

## 3. Results

### 3.1. Background Characteristics of the Studied Patients

A total of 398 HIV‐positive patients were included in the study, yielding a response rate of 95.2%. Females outnumbered males (*n* = 266; 66.8%), with a male‐to‐female ratio of 1:2. Patients’ ages ranged from 18 to 85 years, with a median (IQR) of 50 (42–56) years. Of these, one‐third (134; 33.7%) are aged between 46 and 55 years (Table 1).

**TABLE 1:** Background characteristics of HIV‐positive patients attending the ART clinic at Tikur Anbessa Specialized Hospital and Yekatit 12 Hospital Medical College, Addis Ababa, Ethiopia (*n* = 398).

Variable	Frequency	Percent (%)
*Age category*		
18–35 years	54	13.6
36–45 years	101	25.4
46–55 years	134	33.7
> 55 years	109	27.4
Median (interquartile range)	50 (42–56)	

*Sex*		
Male	132	33.2
Female	266	66.8

*Marital status*		
Never married	86	21.6
Married	141	35.4
Divorced/separated	79	19.8
Widowed	92	23.1

*Educational level*		
No formal education	55	13.8
Primary education	106	26.6
Secondary education	137	34.4
Tertiary education	100	25.1

*Employment status*		
Unemployed	146	36.7
Self‐employed	141	35.4
Employed	111	27.9

*Note:* ART, antiretroviral therapy.

Abbreviation: HIV, human immunodeficiency virus.

With regard to marital status, 101 (25.4%) were married, 92 (23.1%) widowed, 86 (21.6%) never married, and 79 (19.8%) divorced/separated. Regarding educational level, 137 (34.4%) had secondary education, 106 (26.6%) primary, 100 (25.1%) tertiary, and 55 (13.8%) no formal education. With regard to employment status, 146 (36.7%) were unemployed, 141 (35.4%) self‐employed, and 111 (27.9%) employed (Table 1).

### 3.2. Clinical Profile of the Studied Patients

The median duration since HIV diagnosis was 19 years (IQR: 15–20), with most patients (77.4%) diagnosed over 15 years ago; similarly, the median duration since ART initiation was 17 years (IQR: 14–20), with 72.4% on ART for over 15 years. The predominant regimen was TDF + 3TC + DTG (*n* = 288; 72.8%), followed by TDF + 3TC + ATV/r (*n* = 46; 11.6%) and AZT + 3TC + ATV/r (*n* = 20; 5.0%). A history of ART change was documented in 364 (91.5%) of the patients. Among these, 69.2% (*n* = 252) were due to updated treatment guidelines, 25.3% (*n* = 92) to treatment failure, 3.3% (*n* = 12) to drug toxicity, and 2.2% (*n* = 8) were unspecified. Hypertension was the most common comorbidity (*n* = 110; 27.6%), followed by diabetes mellitus (*n* = 60; 15.1%). Only 39 (9.8%) were receiving statin therapy at the time of data collection. Most patients had CD4 counts of 200–500 cells/mm^3^ (*n* = 237; 59.5%) and viral suppression < 50 copies/mL (*n* = 374; 94.0%) (Table 2).

**TABLE 2:** Clinical characteristics of HIV‐positive patients attending the ART clinic at Tikur Anbessa Specialized Hospital and Yekatit 12 Hospital Medical College, Addis Ababa, Ethiopia (*n* = 398).

Variable	Frequency	Percent (%)
*Duration since diagnosis*		
< 5 years	8	2.0
5–10 years	46	11.6
11–15 years	36	9.0
> 15 years	308	77.4
Median (interquartile range)	19 (15–20)	

*Duration since ART initiation*		
< 5 years	12	3.0
5–10 years	52	13.1
11–15 years	46	11.6
> 15 years	288	72.4
Median (interquartile range)	17 (14–20)	

*ART regimen*		
TDF + 3TC + DTG	288	72.8
TDF + 3TC + ATV/r	46	11.6
AZT + 3TC + ATV/r	20	5.0
TDF + 3TC + EFV	16	4.0
DRV/r + ABC + 3TC + 3TC	10	2.5
DRV/r + DTG + AZT + 3TC	8	2.0
ABC + 3TC + LPV/r	4	1.0
DRV/r + DTG + TDF + 3TC	4	1.0
ABC + 3TC + EFV	2	0.5

*History of ART change*		
No	34	8.5
Yes	364	91.5

*Reason for ART change*		
Updated guideline	252	69.2
Treatment failure	92	25.3
Drug toxicity	12	3.3
Unspecified	8	2.2

*Comorbidities*		
None		
Hypertension	110	27.6
Diabetes mellitus	60	15.1
Cardiac disease	16	4.0
Chronic kidney disease	10	2.5
Neurologic disorders	7	1.8
Thyroid disorders	6	1.5
Malignancy	4	1.0
Asthma	4	1.0
Chronic liver disease	4	1.0
Others	2	0.5

*Statin use*		
No	359	90.2
Yes	39	9.8

*CD4 count*		
< 200 cells/mm^3^	25	6.3
200–500 cells/mm^3^	237	59.5
> 500 cells/mm^3^	136	34.2
Median (interquartile range)	425 (313–562)	

*Viral load*		
< 50 copies/mL	374	94.0
≥ 50 copies/mL	24	6.0

*Note:* ART: antiretroviral therapy; ATV/r: atazanavir/ritonavir; AZT: zidovudine (azidothymidine); DTG: dolutegravir; EFV: efavirenz; LPV/r: lopinavir/ritonavir; 3TC: lamivudine.

Abbreviations: CD4, cluster of differentiation 4; HIV, human immunodeficiency virus; PIs, protease inhibitors; TDF, tenofovir disoproxil fumarate.

### 3.3. Behavioral and Lifestyle Details of the Studied Patients

Nearly all patients (381; 95.7%) had never smoked cigarettes, with only 17 (4.3%) being former smokers. The majority never consumed alcohol (*n* = 321; 80.7%), while 77 (19.3%) reported occasional use. Regarding physical activity, the majority had moderate activity (*n* = 308; 76.9%), followed by high activity (*n* = 48; 12.1%) and a sedentary lifestyle (*n* = 44; 11.1%) (Table 3).

**TABLE 3:** Behavioral and lifestyle details of HIV‐positive patients attending the ART clinic at Tikur Anbessa Specialized Hospital and Yekatit 12 Hospital Medical College, Addis Ababa, Ethiopia (*n* = 398).

Variable	Frequency	Percent (%)
*Body mass index*		
Underweight (< 18.5)	37	9.3
Normal (18.5–24.9)	234	58.8
Overweight (25–29.9)	101	25.4
Obese (≥ 30)	26	6.5
Median (interquartile range)	23.21 (20.6–26.1)	

*Cigarette smoking status*		
Never	381	95.7
Former smoker	17	4.3

*Alcohol consumption*		
Never	321	80.7
Occasional	77	19.3

*Physical activity*		
Sedentary	44	11.1
Moderate	308	76.9
High	48	12.1

*Note:* ART: antiretroviral therapy.

Abbreviation: HIV, human immunodeficiency virus.

### 3.4. Prevalence of Dyslipidemia Among HIV‐Positive Patients

**FIGURE 1:**
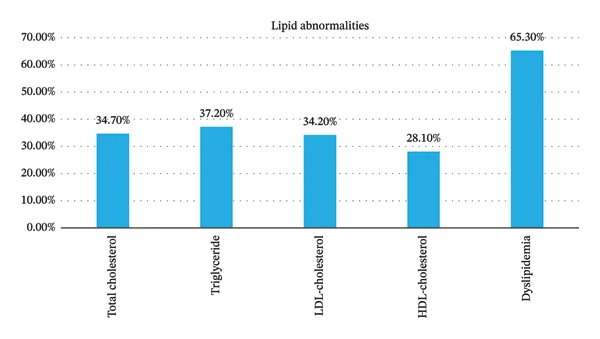
Prevalence of dyslipidemia among HIV‐positive patients attending the ART clinic at Tikur Anbessa Specialized Hospital and Yekatit 12 Hospital Medical College, Addis Ababa, Ethiopia (*n* = 398). Abbreviations: HDL‐C: high‐density lipoprotein cholesterol; LDL‐C: low‐density lipoprotein cholesterol.

Out of the total, dyslipidemia was present in 260 (65.33%; 95% CI: 60.63–70.02) patients. Among the lipid components, total cholesterol was elevated in 138 (34.7%) patients, TG were high in 148 (37.2%), LDL‐cholesterol was high in 136 (34.2%), and HDL‐cholesterol was deranged in 112 (28.1%) patients (Figure 1).

### 3.5. Factors Associated With Dyslipidemia Among HIV‐Positive Patients

In this study, patients with prior ART regimen switches had significantly higher odds of dyslipidemia compared to those without (AOR = 2.62; 95% CI: 1.13–6.04). Patients with hypertension had increased odds of dyslipidemia compared to those without (AOR = 2.45; 95% CI: 1.28–4.69). Furthermore, BMI categories demonstrated a positive relationship with dyslipidemia. Overweight patients had higher odds (AOR = 3.56; 95% CI: 1.44–8.81), while obese patients showed markedly increased odds compared to underweight patients (AOR = 5.15; 95% CI: 1.16–22.82). Finally, physical activity level had a highly significant effect on dyslipidemia. Sedentary patients had substantially higher odds (AOR = 9.46; 95% CI: 2.46–36.32), and moderately active patients also showed elevated odds compared to those with high activity (AOR = 3.80; 95% CI: 1.77–8.18) (Table 4).

**TABLE 4:** Factors associated with dyslipidemia among HIV‐positive patients attending the ART clinic at Tikur Anbessa Specialized Hospital and Yekatit 12 Hospital Medical College, Addis Ababa, Ethiopia (*n* = 398).

Variable	Dyslipidemia		COR (95% CI)	AOR (95% CI)	*p* value
	Present (%)	Absent (%)			
*Age category*					
18–35 years	23 (42.6)	31 (57.4)	1	1	—
36–45 years	64 (63.4)	37 (36.6)	2.33 (1.19, 4.58)	1.29 (0.59, 2.83)	0.520
46–55 years	92 (68.7)	42 (31.3)	2.95 (1.54, 5.66)	1.23 (0.58, 2.59)	0.594
> 55 years	81 (74.3)	28 (25.7)	3.90 (1.96, 7.77)	1.21 (0.52, 2.80)	0.652

*ART regimen*					
First line	196 (64.1)	110 (35.9)	1.48 (0.62, 3.55)	1.73 (0.54, 5.52)	0.352
Second line	52 (74.3)	18 (25.7)	2.41 (0.89, 6.52)	2.93 (0.829, 10.37)	0.095
Third line	12 (54.5)	10 (45.5)	1	1	—

*History of ART change*					
No	14 (41.2)	20 (58.8)	1	1	—
Yes	246 (67.6)	118 (32.4)	2.98 (1.45, 6.10)	2.62 (1.13, 6.04)	**0.024**

*Hypertension status*					
No	172 (59.7)	116 (40.3)	1	1	—
Yes	88 (80.0)	22 (20.0)	2.70 (1.60, 4.55)	2.45 (1.28, 4.69)	**0.007**

*Diabetes mellitus*					
No	214 (63.3)	124 (36.7)	1	1	—
Yes	46 (76.7)	14 (23.3)	1.90 (1.01, 3.60)	1.91 (0.84, 4.30)	0.120

*Cardiac disease*					
No	246 (64.4)	136 (35.6)	1	1	—
Yes	14 (87.5)	2 (12.5)	3.87 (0.99, 17.28)	1.34 (0.26, 6.91)	0.731

*Viral load*					
< 50 copies/mL	240 (64.2)	134 (35.8)	1	1	—
≥ 50 copies/mL	20 (83.3)	4 (16.7)	2.79 (0.94, 8.34)	1.78 (0.51, 6.17)	0.365

*Body mass index*					
Underweight	15 (40.5)	22 (59.5)	1	1	—
Normal	142 (60.7)	92 (39.3)	2.26 (1.12, 4.59)	1.87 (0.86, 4.07)	0.116
Overweight	80 (79.2)	21 (20.8)	5.59 (2.48, 12.60)	3.56 (1.44, 8.81)	**0.006**
Obese	23 (88.5)	3 (11.5)	11.24 (2.86, 44.27)	5.15 (1.16, 22.82)	**0.031**

*Cigarette smoking status*					
Never	245 (64.3)	136 (35.7)	1	1	—
Former smoker	15 (88.2)	2 (11.8)	4.16 (0.94, 18.48)	2.28 (0.46, 11.22)	0.310

*Alcohol consumption*					
Never	201 (62.6)	120 (37.4)	1	1	—
Occasional	59 (76.6)	18 (23.4)	1.96 (1.10, 3.48)	1.30 (0.66, 2.56)	0.454

*Physical activity*					
Sedentary	40 (90.9)	4 (9.1)	20.00 (6.08, 65.75)	9.46 (2.46, 36.32)	**0.001**
Moderate	204 (66.7)	102 (33.3)	4.00 (2.10, 7.63)	3.80 (1.77, 8.18)	**< 0.001**
High	16 (33.3)	32 (66.7)	1	1	—

*Note:* Only variables with *p* value < 0.25 in the bivariable regression are depicted here. 1: Reference category. Bold values indicate statistical significance.

## 4. Discussion

The purpose of this study was to assess the prevalence of dyslipidemia and associated factors among HIV‐positive patients at selected public hospitals in Addis Ababa, Ethiopia, 2025. The results demonstrated a high prevalence of dyslipidemia (65.3%) in this cohort. Additionally, analyses revealed that history of ART change, hypertension, higher BMI, and lower physical activity levels were independently associated with dyslipidemia. Our findings provide contemporary evidence from a multihospital urban ART population and extend earlier work by assessing a broader set of clinical and behavioral variables.

The dyslipidemia prevalence of 65.3% observed here is comparable to the 63.9% reported from Northwest Ethiopia referral hospitals and exceeds the 55.2% reported at Zewditu Memorial Hospital among ART‐treated patients [22, 30]. Conversely, it is slightly lower than the 74.8% observed at Armed Forces Comprehensive Hospital and 73.7% in ART‐treated patients at Debre Tabor Hospital, Northwest Ethiopia [31, 32]. Notably, our prevalence is lower than rates reported in Northwest Ethiopia (73.8%), Eritrea (86.6%), Uganda (78.0%), Rwanda (82.7%), and China (75.5%) [15, 27, 29, 33, 34]. These differences likely reflect variations in patient demographics (age and sex), ART era and regimen composition (particularly use of dyslipidemia‐prone ART agents such as PIs and NRTIs), diagnostic criteria (guideline cutoffs), and laboratory methods (fasting vs. nonfasting). For example, the Rwandan study used stricter local thresholds with lower LDL‐C cutoffs [34], which can increase the classification of dyslipidemia relative to our use of the ESC/EAS criteria. This finding is supported by the recent Italian study reporting that only 27.2% of Italian PLHIV attained recommended LDL‐C targets despite being on lipid‐lowering agents [11].

Several factors may explain the high dyslipidemia burden in our cohort. The cohort had long ART exposure (median duration 17 years) and frequent regimen switches (91.5%), with many switches to PIs such as ATV/r. The predominance of older adults (median age 50 years) and history of PI exposure distinguish this cohort from ART‐naïve or shorter‐duration cohorts, emphasizing cumulative ART toxicity over viral effects alone [23].

Methodological differences also matter: We applied the ESC/EAS guidelines [16] and used fasting lipid samples, whereas some prior Ethiopian studies used NCEP ATP III cutoffs [13, 14]. Fasting triglyceride measurements yield lower, more stable values than nonfasting measurements, which can overestimate TG by about 20–50 mg/dL due to postprandial lipemia [35]; our fasting protocol thus likely improved specificity for true hypertriglyceridemia compared with routine clinic testing.

In this study, history of ART change was associated with dyslipidemia; patients with prior ART regimen switches had significantly higher odds of dyslipidemia compared to those without (AOR = 2.62, 95% CI: 1.13–6.04). This is in agreement with earlier findings from Togo [36], where ART regimen substitution was independently associated with any form of dyslipidemia. This finding may warrant deeper exploration to identify the definitive culprit. Among others, the observed association may reflect multiple factors, including treatment failure, toxicity‐driven switches, or transitions to newer regimens such as dolutegravir‐based therapy, all of which have been associated with metabolic alterations in PLHIV [15, 37].

Additionally, it can be postulated that switching often occurs in metabolically compromised subgroups, including lipodystrophy. This is supported by the older adult constitution of the cohort (median age 50 years) and female predominance (66.8%), which may compound postmenopausal lipotoxicity [38]. Moreover, sequential exposure through switches likely exposes patients to multiple PI “hits” (in this study, 91.5% switch rate and 23.1% PI history), which irreversibly inhibit adipocyte differentiation [3, 4].

This study demonstrated that patients with hypertension had increased odds of dyslipidemia compared to normotensive patients (AOR = 2.45, 95% CI: 1.28–4.69). This is supported by previous independent observations [22, 39]. Mechanistically, multiple pathways explain this association. For instance, elevated blood pressure induces oxidative stress and vascular inflammation, which impairs HDL functionality and promotes LDL oxidation, both hallmarks of this cohort’s lipid profile (28.1% hypoalphalipoproteinemia, 34.2% hyperbetalipoproteinemia) [40]. Additionally, renin–angiotensin–aldosterone system (RAAS) overactivation in hypertension upregulates hepatic enzymes that promote cholesterol synthesis while suppressing LDL receptor expression, driving hypercholesterolemia. Angiotensin II directly stimulates VLDL production, explaining the 34.7% hypercholesterolemia prevalence in the present study population [41].

In agreement with previous studies [14, 36], BMI category showed a dose–response relationship with dyslipidemia among HIV‐positive individuals, with overweight patients demonstrating nearly threefold higher odds (AOR = 3.56, 95% CI: 1.44–8.81) and obese patients showing fivefold higher odds (AOR = 5.15, 95% CI: 1.16–22.82) compared to underweight patients. Adipose tissue expansion in overweight/obese patients increases free fatty acids, inducing hepatic insulin resistance and VLDL overproduction, resulting in hypertriglyceridemia [42]. This mechanism is plausible for this study, as 37.2% of patients had hypertriglyceridemia. Another mechanism behind this association is that obesity reduces adiponectin (antilipogenic) while elevating leptin and proinflammatory cytokines (TNF‐α and IL‐6), impairing lipoprotein lipase (LPL) activity and HDL‐C clearance, which explains the 28.1% low HDL‐C prevalence observed in this study. Moreover, the long‐term NRTIs (TDF + 3TC in 84.4%) + PI exposure (23.1%) observed in this study exacerbate central adiposity via mitochondrial toxicity and lipodystrophy [43].

In line with earlier studies [36, 44], moreover, this study showed that physical activity level was associated with dyslipidemia, such that moderate activity and sedentary patients had four‐to fifteen‐fold higher odds compared to those with high activity (AOR = 3.80; 95% CI: 1.77–8.18; AOR = 9.46; 95% CI: 2.46–36.32, respectively). This can be explained by the fact that an inactive lifestyle impairs LPL expression in skeletal muscle and adipose tissue, thereby reducing triglyceride clearance from VLDL and chylomicrons. In contrast, exercise enhances muscle fatty acid oxidation, which in turn reduces ectopic lipid deposition in the liver and muscle that induces insulin resistance. Consequently, sedentary patients accumulate visceral fat despite stable BMI, further amplifying dyslipidemia [7].

Furthermore, this multicenter study across two major tertiary hospitals in Addis Ababa thus enhances generalizability to public ART clinics in the study setting. Dyslipidemia was defined using standardized ESC guidelines criteria with fasting lipid samples, which ensures comparability with global literature. Notably, this approach differs from some previous Ethiopian studies that used NCEP ATP III cutoffs; our use of a more stringent LDL‐C threshold aligned with contemporary ESC/EAS guidance may partly explain differences in prevalence estimates across studies. However, findings should be interpreted considering the following limitations. Our findings are based on patients attending two tertiary hospitals in Addis Ababa and may not be generalizable to all PLHIV in Ethiopia, particularly those in rural or primary‐care settings. The cross‐sectional design precludes establishing temporality (e.g., whether elevated BMI preceded dyslipidemia) and capturing longitudinal lipid trajectories after ART regimen changes. Additionally, cardiovascular risk category was not assessed for each patient, which limited further analysis against target lipid levels. Retrospective clinical data introduce risks of recall bias for behavioral factors and incomplete medical records. A significant proportion (9.8%) of the patients were receiving statin therapy at the time of data collection, which may have influenced individual lipid values and potentially led to underestimation of dyslipidemia prevalence. Similarly, self‐reported physical activity and alcohol intake are susceptible to recall bias and social desirability bias, potentially leading to misclassification. Finally, absent pre‐ART lipid profiles, distinguishing ART‐induced from HIV‐related dyslipidemia contributions was not feasible.

## 5. Conclusions and Recommendations

This study found a dyslipidemia prevalence of 65.3% among HIV‐positive adults attending ART clinics at the study hospitals. Significant associated factors included prior ART regimen change, hypertension, elevated BMI, and low physical activity. Clinicians should prioritize routine lipid profile screening for all patients on ART, with targeted monitoring in high‐risk subgroups. Multidisciplinary care coordination is essential to manage cardiometabolic comorbidities alongside HIV. Local health authorities should incorporate dyslipidemia screening into national ART guidelines and provide resources for point‐of‐care lipid testing at Ethiopian ART clinics. Finally, future studies should employ longitudinal designs to elucidate causal pathways between ART switches and dyslipidemia, incorporating PI exposure duration and genetic factors, while also integrating objective measures of physical activity, such as accelerometry. They should also assess individual cardiovascular risk scores to enable analysis against target lipid levels.

**Nomenclature**
3TC: Lamivudine
ART: Antiretroviral therapy
ATV/r: Atazanavir/ritonavir
AZT: Zidovudine (azidothymidine)
BMI: Body mass index
CD4: Cluster of differentiation 4
CVD: Cardiovascular disease
DTG: Dolutegravir
ESC: European Society of Cardiology
EFV: Efavirenz
HDL‐C: High‐density lipoprotein cholesterol
HIV: Human immunodeficiency virus
IQR: Interquartile range
LDL‐C: Low‐density lipoprotein cholesterol
LPV/r: Lopinavir/ritonavir
PIs: Protease inhibitors
PLHIV: People living with HIV
SPSS: Statistical Package for Social Science
SSA: Sub‐Saharan Africa
TASH: Tikur Anbessa Specialized Hospital
TC: Total cholesterol
TDF: Tenofovir disoproxil fumarate
TG: Triglycerides
VLDL: Very low‐density lipoprotein
Y12HMC: Yekatit 12 Hospital Medical College

## Author Contributions

Nebiat Adane conceived and designed the study. Nebiat Adane and Subah Abderehim Yesuf contributed to proposal development, data analysis, interpretation, and manuscript writing. Abraham Tekola Gebremedhn and Mahder Abebe Asres facilitated data collection, while Mekoya Mengistu, Tariku Fekadu, and Nevena Ivanova provided critical revisions to the manuscript. All authors contributed to result interpretation and critically revised the manuscript for important intellectual content.

## Funding

The authors received no specific funding for this work.

## Disclosure

All authors approved the final version for submission and agree to be accountable for all aspects of the work. Parts of this manuscript were submitted as a thesis by NA to Y12HMC but have not been published elsewhere.

## Ethics Statement

Ethical clearance was obtained from the Institutional Review Board of Yekatit 12 Hospital and Medical College (ref. no. RPO/10/2025). A support letter from the Department of Internal Medicine, Y12HMC (ref. no. YH12:337/300/5325), was submitted to the ART unit at each study site prior to data collection. Patients’ records were de‐identified before analysis, and the confidentiality of patients’ records was maintained. Patients approached for missing information provided verbal informed consent prior to interviews. Finally, those identified with clinically significant dyslipidemia were referred to their clinicians for further evaluation and management.

## Conflicts of Interest

The authors declare no conflicts of interest.

## Data Availability

The data that support the findings of this study are available on request from the corresponding author. The data are not publicly available due to privacy and ethical restrictions.
